# Network Completion Using Dynamic Programming and Least-Squares Fitting

**DOI:** 10.1100/2012/957620

**Published:** 2012-11-01

**Authors:** Natsu Nakajima, Takeyuki Tamura, Yoshihiro Yamanishi, Katsuhisa Horimoto, Tatsuya Akutsu

**Affiliations:** ^1^Bioinformatics Center, Institute for Chemical Research, Kyoto University Gokasho, Uji, Kyoto 611-0011, Japan; ^2^Division of System Cohort, Multi-scale Research Center for Medical Science, Medical Institute of Bioregulation, Kyushu University, 3-1-1 Maidashi, Higashi-ku, Fukuoka, Fukuoka 812-8582, Japan; ^3^Computational Biology Research Center, National Institute of Advanced Industrial Science and Technology, 2-4-7 Aomi, Koto-ku, Tokyo 135-0064, Japan

## Abstract

We consider the problem of network completion, which is to make the minimum amount of modifications to a given network so that the resulting network is most consistent with the observed data. We employ here a certain type of differential equations as gene regulation rules in a genetic network, gene expression time series data as observed data, and deletions and additions of edges as basic modification operations. In addition, we assume that the numbers of deleted and added edges are specified. For this problem, we present a novel method using dynamic programming and least-squares fitting and show that it outputs a network with the minimum sum squared error in polynomial time if the maximum indegree of the network is bounded by a constant. We also perform computational experiments using both artificially generated and real gene expression time series data.

## 1. Introduction

Analysis of biological networks is one of the central research topics in computational systems biology. In particular, extensive studies have been done on inference of genetic networks using gene expression time series data, and a number of computational methods have been proposed, which include methods based on Boolean networks [1, 2], Bayesian networks [3, 4], time-delayed Bayesian networks [5], graphical Gaussian models [6–8], differential equations [9, 10], mutual information [11, 12], and linear classification [13]. However, there is not yet an established or standard method for inference of genetic networks, and thus it still remains a challenging problem.

One of the possible reasons for the difficulty of inference is that the amount of available high-quality gene expression time series data is still not enough, and thus it is intrinsically difficult to infer the correct or nearly correct network from such a small amount of data. Therefore, it is reasonable to try to develop another approach. For that purpose, we proposed an approach called network completion [14] by following Occam's razor, which is a well-known principle in scientific discovery. Network completion is, given an initial network and an observed dataset, to modify the network by the minimum amount of modifications so that the resulting network is (most) consistent with the observed data. Since we were interested in inference of signaling networks in our previous study [14], we assumed that activity levels or quantities of one or a few kinds of proteins can only be observed. Furthermore, since measurement errors were considered to be large and we were interested in theoretical analysis of computational complexity and sample complexity, we adopted the Boolean network [15] as a model of signaling networks. We proved that network completion is computationally intractable (NP-hard) even for tree-structured networks. In order to cope with this computational difficulty, we developed an integer linear programming-based method for completion of signaling pathways [16]. However, this method could not handle addition of edges because of its high computational cost.

In this paper, we propose a novel method, DPLSQ, for completing genetic networks using gene expression time series data. Different from our previous studies [14, 16], we employ a model based on differential equations and assume that expression values of all nodes can be observed. DPLSQ is a combination of least-squares fitting and dynamic programming, where least-squares fitting is used for estimating parameters in differential equations and dynamic programming is used for minimizing the sum of least-squares errors by integrating partial fitting results on individual genes under the constraint that the numbers of added and deleted edges must be equal to the specified ones. One of the important features of DPLSQ is that it can output an optimal solution (i.e., minimum squared sum) in polynomial time if the maximum indegree (i.e., the maximum number of input genes to a gene) is bounded by a constant. Although DPLSQ does not automatically find the minimum modification, it can be found by examining varying numbers of added/deleted edges, where the total number of such combinations is polynomially bounded. If a null network (i.e., a network having no edges) is given as an initial network, DPLSQ can work as an inference method for genetic networks.

In order to examine the effectiveness of DPLSQ, we perform computational experiments using artificially generated data. We also make computational comparison of DPLSQ as an inference method with other existing tools using artificial data. Furthermore, we perform computational experiments on DPLSQ using real cell cycle expression data of * Saccharomyces cerevisiae*.

## 2. Method

**Figure 1:**
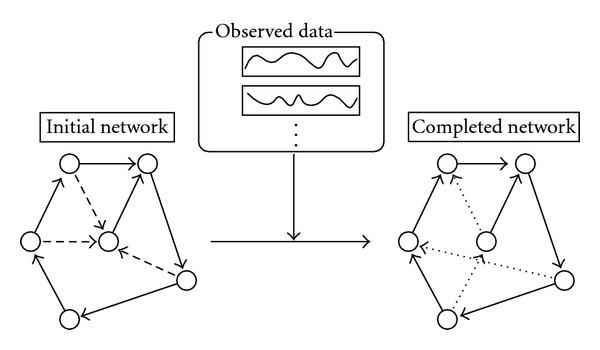
Network completion by addition and deletion of edges. Dashed edges and dotted edges denote deleted edges and added edges, respectively.

The purpose of network completion is to modify a given network with the minimum number of modifications so that the resulting network is most consistent with the observed data. In this paper, we consider additions and deletions of edges as modification operations (see Figure 1). If we begin with a network with an empty set of edges, it corresponds to network inference. Therefore, network completion includes network inference although it may not necessarily work better than the existing methods if applied to network inference.

In the following, *G*(*V*, *E*) denotes a given network where *V* and *E* are the sets of nodes and directed edges respectively, where each node corresponds to a gene and each edge represents some direct regulation between two genes. Self loops are not allowed in *E* although it is possible to modify the method so that self-loops are allowed. In this paper, *n* denotes the number of genes (i.e., the number of nodes) and we let *V* = {*v*_1_,…, *v*_*n*_}. For each node *v*_*i*_, *e* ^−^(*v*_*i*_) and deg^−^(*v*_*i*_), respectively, denote the set of incoming edges to *v*_*i*_ and the number of incoming edges to *v*_*i*_ as defined follows:

(1) $$\begin{array}{c} e^{-}\left(v_{i}\right)=\left\{v_{j}\;|\;\left(v_{j},v_{i}\right)\in E\right\},\\ \text{de}{\text{g}}^{-}\left(v_{i}\right)=\left|e^{-}\left(v_{i}\right)\right|. \end{array}$$

DPLSQ consists of two parts: (i) parameter estimation and (ii) network structure inference. We employ least-squares fitting for the former part and dynamic programming for the latter part. Furthermore, there are three variants on the latter parts: (a) completion by addition of edges, (b) completion by deletion of edges, and (c) completion by addition and deletion of edges. Although the last case includes the first and second cases, we explain all of these for the sake of simplicity of explanation.

### 2.1. Model of Differential Equation and Estimation of Parameters

**Figure 2:**
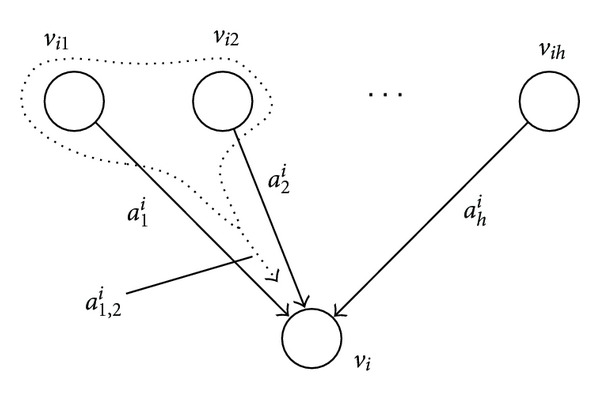
Dynamics model for a node.

We assume that dynamics of each node *v*_*i*_ is determined by a differential equation:

(2) $$\begin{array}{c} \frac{dx_{i}}{dt}=a_{0}^{i}+\sum_{j=1}^{h}a_{j}^{i}x_{i_{j}}+\sum_{j<k}a_{j,k}^{i}x_{i_{j}}x_{i_{k}}+b^{i}\omega , \end{array}$$

where *v*_*i*_1__,…, *v*_*i*_*h*__ are incoming nodes to *v*_*i*_, *x*_*i*_ corresponds to the expression value of the *i*th gene, and *ω* denotes a random noise. The second and third terms of the right-hand side of the equation represent linear and nonlinear effects to node *v*_*i*_, respectively (see Figure 2), where positive *a*_*j*_^*i*^ or *a*_*j*,*k*_^*i*^ corresponds to an activation effect and negative *a*_*j*_^*i*^ or *a*_*j*,*k*_^*i*^ corresponds to an inhibition effect.

In practice, we replace derivative by difference and ignore the noise term as follows:

(3) $$\begin{array}{c} x_{i}\left(t+1\right)=x_{i}\left(t\right)+\Delta t\left(a_{0}^{i}+\sum_{j=1}^{h}a_{j}^{i}x_{i_{j}}\left(t\right)+\sum_{j<k}a_{j,k}^{i}x_{i_{j}}\left(t\right)x_{i_{k}}\left(t\right)\right), \end{array}$$

where Δ*t* denotes the time step.

We assume that time series data *y*_*i*_(*t*)s, which correspond to *x*_*i*_(*t*)s, are given for *t* = 0,1,…, *m*. Then, we can use the standard least-squares fitting method to estimate the parameters *a*_*j*_^*i*^s and *a*_*j*,*k*_^*i*^s.

In applying the least-squares fitting method, we minimize the following objective function:

(4) Si1,i2,…,ihi=∑t=1m|yi(t+1)    −[yi(t)+Δt(a0i+∑j=1hajiyij(t)+∑j<kaj,kiyij(t)yik(t))]|2.

### 2.2. Completion by Addition of Edges

 In this subsection, we consider the problem of adding *k* edges in total so that the sum of least-squares errors is minimized.

Let *σ*_*k*_*j*_,*j*_^+^ denote the minimum least-squares error when adding *k*_*j*_ edges to the *j*th node, which is formally defined by

(5) $$\begin{array}{c} \sigma_{k_{j},j}^{+}=\underset{j_{1},j_{2},\ldots ,j_{k_{j}}}{\min}S_{j_{1},j_{2},\ldots ,j_{k_{j}}}^{j}, \end{array}$$

where each *v*_*j*_*l*__ must be selected from *V* − *v*_*j*_ − *e* ^−^(*v*_*j*_). In order to avoid combinatorial explosion, we constrain the maximum *k* to be a small constant *K* and let *σ*_*k*_*j*_,*j*_^+^ = +*∞* for *k*_*j*_ > *K* or *k*_*j*_ + deg^−^(*v*_*j*_) ≥ *n*. Then, the problem is stated as

(6) $$\begin{array}{c} \underset{k_{1}+k_{2}+\cdots +k_{n}=k}{\min}\;\sum_{j=1}^{n}\sigma_{k_{j},j}^{+}. \end{array}$$

Here, we define *D* ^+^[*k*, *i*] by

(7) $$\begin{array}{c} D^{+}\left[k,i\right]=\underset{k_{1}+k_{2}+\cdots +k_{i}=k}{\min}\;\sum_{j=1}^{i}\sigma_{k_{j},j}^{+}. \end{array}$$

Then, *D* ^+^[*k*, *n*] is the objective value (i.e., the minimum of the sum of least-squares errors when adding *k* edges).

The entries of *D* ^+^[*k*, *j*] can be computed by the following dynamic programming algorithm:

(8) $$\begin{array}{c} D^{+}\left[k,1\right]=\sigma_{k,1}^{+},\\ D^{+}\left[k,j+1\right]=\underset{k^{\prime }+k^{\prime \prime }=k}{\min}\left\{D^{+}\left[k^{\prime },j\right]+\sigma_{k^{\prime \prime },j+1}^{+}\right\}. \end{array}$$

It is to be noted that *D* ^+^[*k*, *n*] is determined uniquely regardless of the ordering of nodes in the network. The correctness of this dynamic programming algorithm can be seen by

(9) min⁡k1+k2+⋯+kn=k∑j=1nσkj,j+=min⁡k′+k′′=k{min⁡k1+k2+⋯+kn−1=k′∑j=1n−1σkj,j++σk′′,n+}=min⁡k′+k′′=kD+[k′,n−1]+σk′′,n+.

### 2.3. Completion by Deletion of Edges

 In the above, we considered network completion by addition of edges. Here, we consider the problem of deleting *h* edges in total so that the sum of least-squares errors is minimized.

Let *σ*_*h*_*j*_,*j*_^−^ denote the minimum least-squares error when deleting *h*_*j*_ edges from the set *e* ^−^(*v*) of incoming edges to *v*_*j*_. As in Section 2.2, we constrain the maximum *h*_*j*_ to be a small constant *H* and let *σ*_*h*_*j*_,*j*_^−^ = +*∞* if *h*_*j*_ > *H* or deg^−^(*v*_*j*_) − *h*_*j*_ < 0. Then, the problem is stated as

(10) $$\begin{array}{c} \underset{h_{1}+h_{2}+\cdots +h_{n}=h}{\min}\;\sum_{j=1}^{n}\sigma_{k_{j},j}^{-}. \end{array}$$

Here, we define *D* ^−^[*k*, *i*] by

(11) $$\begin{array}{c} D^{-}\left[k,i\right]=\underset{k_{1}+k_{2}+\cdots +k_{i}=k}{\min}\;\sum_{j=1}^{i}\sigma_{k_{j},j}^{-}. \end{array}$$

Then, we can solve network completion by deletion of edges using the following dynamic programming algorithm:

(12) $$\begin{array}{c} D^{-}\left[k,1\right]=\sigma_{k,1}^{-},\\ D^{-}\left[k,j+1\right]=\underset{k^{\prime }+k^{\prime \prime }=k}{\min}\left\{D^{-}\left[k^{\prime },j\right]+\sigma_{k^{\prime \prime },j+1}^{-}\right\}. \end{array}$$

### 2.4. Completion by Addition and Deletion of Edges

We can combine the above two methods into network completion by addition and deletion of edges.

Let *σ*_*h*_*j*_,*k*_*j*_,*j*_ denote the minimum least-squares error when deleting *h*_*j*_ edges from *e* ^−^(*v*_*j*_) and adding *k*_*j*_ edges to *e* ^−^(*v*_*j*_) where deleted and added edges must be disjoint. We constrain the maximum *h*_*j*_ and *k*_*j*_ to be small constants *H* and *K*. We let *σ*_*h*_*j*_,*k*_*j*_,*j*_ = +*∞* if *h*_*j*_ > *H*, *k*_*j*_ > *K*, *k*_*j*_ − *h*_*j*_ + deg^−^(*v*_*j*_) ≥ *n*, or *k*_*j*_ − *h*_*j*_ + deg^−^(*v*_*j*_) < 0 holds. Then, the problem is stated as

(13) $$\begin{array}{c} \underset{\begin{array}{c} h_{1}+h_{2}+\cdots +h_{n}=h\\ k_{1}+k_{2}+\cdots +k_{n}=k \end{array}}{\min}\;\sum_{j=1}^{n}\sigma_{h_{j},k_{j},j}. \end{array}$$

Here, we define *D*[*h*, *k*, *i*] by

(14) D[h,k,i]min⁡h1+h2+⋯+hi=hk1+k2+⋯+ki=k ∑j=1iσhj,kj,j.

Then, we can solve network completion by addition and deletion of edges using the following dynamic programming algorithm:

(15) $$\begin{array}{c} D\left[h,k,1\right]=\sigma_{h,k,1},\\ D\left[h,k,j+1\right]=\underset{\begin{array}{c} h^{\prime }+h^{\prime \prime }=h\\ k^{\prime }+k^{\prime \prime }=k \end{array}}{\min}\left\{D\left[h^{\prime },k^{\prime },j\right]+\sigma_{h^{\prime \prime },k^{\prime \prime },j+1}\right\}. \end{array}$$

### 2.5. Time Complexity Analysis

 In this subsection, we analyze the time complexity of DPLSQ. Since completion by addition of edges and completion by deletion of edges are special cases of completion by addition and deletion of edges, we focus on completion by addition and deletion of edges.

First, we analyze the time complexity required per least-squares fitting. It is known that least-squares fitting for linear systems can be done in *O*(*mp* ^2^ + *p* ^3^) time where *m* is the number of data points and *p* is the number of parameters. Since our model has *O*(*n* ^2^) parameters, the time complexity is *O*(*mn* ^4^ + *n* ^6^). However, if we can assume that the maximum indegree in a given network is bounded by a constant, the number of parameters is bounded by a constant, where we have already assumed that *H* and *K* are constants. In this case, the time complexity for least-squares fitting can be estimated as *O*(*m*).

Next, we analyze the time complexity required for computing *σ*_*h*_*j*_,*k*_*j*_,*j*_. In this computation, we need to examine combinations of deletions of *h*_*j*_ edges and additions of *k*_*j*_ edges. Since *h*_*j*_ and *k*_*j*_ are, respectively, bounded by constants *H* and *K*, the number of combinations is *O*(*n* ^*H*+*K*^). Therefore, the computation time required per *σ*_*h*_*j*_,*k*_*j*_,*j*_ is *O*(*n* ^*H*+*K*^(*mn* ^4^ + *n* ^6^)) including the time for least-squares fitting. Since we need to compute *σ*_*h*_*j*_,*k*_*j*_,*j*_ for *H* × *K* × *n* combinations, the total time required for computation of *σ*_*h*_*j*_,*k*_*j*_,*j*_s is *O*(*n* ^*H*+*K*+1^(*mn* ^4^ + *n* ^6^)).

Finally, we analyze the time complexity required for computing *D*[*h*, *k*, *i*]s. We note that the size of table *D*[*h*, *k*, *i*] is *O*(*n* ^3^), where we are assuming that *h* and *k* are *O*(*n*). In order to compute the minimum value for each entry in the dynamic programming procedure, we need to examine (*H* + 1)(*K* + 1) combinations, which is *O*(1). Therefore, the computation time required for computing *D*[*h*, *k*, *i*]s is *O*(*n* ^3^). Since this value is clearly smaller than the one for *σ*_*h*_*j*_,*k*_*j*_,*j*_s, the total time complexity is

(16) $$\begin{array}{c} O\left(n^{H+K+1}\cdot \left(mn^{4}+n^{6}\right)\right). \end{array}$$

Although this value is too high, it can be significantly reduced if we can assume that the maximum degree of an input network is bounded by a constant. In this case, the least-squares fitting can be done in *O*(*m*) time per execution. Furthermore, the number of combinations of deleting at most *h*_*j*_ edges is bounded by a constant. Therefore, the time complexity required for computing *σ*_*h*_*j*_,*k*_*j*_,*j*_s is reduced to *O*(*mn* ^*K*+1^). Since the time complexity for computing *D*[*h*, *k*, *i*]s remains *O*(*n* ^3^), the total time complexity is

(17) $$\begin{array}{c} O\left(mn^{K+1}+n^{3}\right). \end{array}$$

This number is allowable in practice if *K* ≤ 2 and *n* is not too large (e.g., *n* ≤ 100).

## 3. Results

We performed computational experiments using both artificial data and real data. All experiments on DPLSQ were performed on a PC with Intel Core i7-2630QM CPU (2.00 GHz) with 8 GB RAM running under the Cygwin on Windows 7. We employed the liblsq library (http://www2.nict.go.jp/aeri/sts/stmg/K5/VSSP/install_lsq.html) for a least-squares fitting method.

### 3.1. Completion Using Artificial Data

 In order to evaluate the potential effectiveness of DPLSQ, we began with network completion using artificial data. To our knowledge, there is no available tool that performs the same task. Although some of the existing inference methods employ incremental modifications of networks, the number of added/deleted edges cannot be specified. Therefore, we did not compare DPLSQ for network completion with other methods (but we compared it with the existing tools for network inference).

**Figure 3:**
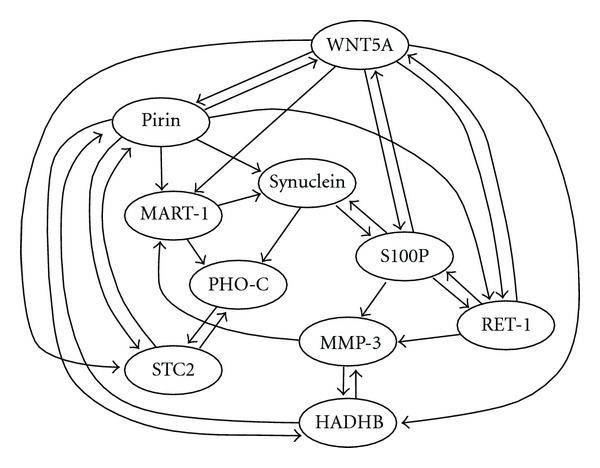
Structure of WNT5A network [17].

We employed the structure of the real biological network named WNT5A (see Figure 3) [17]. For each node *v*_*i*_ with *h* input nodes, we considered the following model:

(18) $$\begin{array}{c} x_{i}\left(t+1\right)=x_{i}\left(t\right)+\Delta t\left(a_{0}^{i}+\sum_{j=1}^{h}a_{j}^{i}x_{i_{j}}+\sum_{j<k}a_{j,k}^{i}x_{i_{j}}\left(t\right)x_{i_{k}}\left(t\right)+b_{i}\omega \right), \end{array}$$

where *a*_*j*_^*i*^s and *a*_*j*,*k*_^*i*^s are constants selected uniformly at random from [−1,1] and [−0.5,0.5], respectively. The reason why the domain of *a*_*j*,*k*_^*i*^s is smaller than that for *a*_*j*_^*i*^s is that non-linear terms are not considered as strong as linear terms. It should also be noted that *b*_*i*_ *ω* is a stochastic term, where *b*_*i*_ is a constant (we used *b*_*i*_ = 0.2 in all computational experiments) and *ω* is a random noise taken uniformly at random from [−1,1].

For artificial generation of observed data *y*_*i*_(*t*), we used

(19) $$\begin{array}{c} y_{i}\left(t\right)=x_{i}\left(t\right)+o^{i}\epsilon , \end{array}$$

where *o* ^*i*^ is a constant denoting the level of observation errors and *ϵ* is a random noise taken uniformly at random from [1, −1]. Since the use of time series data beginning from only one set of initial values easily resulted in overfitting, we generated time series data beginning from 20 sets of initial values taken uniformly at random from [1, −1], where the number of time points for each set was set to 10 and Δ*t* = 0.2 was used as the period between the consecutive two time points. Therefore, 20 sets of time series data, each of which consisted of 10 time points, were used per trial (200 time points were used in total per trial). It is to be noted that in our preliminary experiments, the use of too small Δ*t* resulted in too small changes of expression values whereas the use of large Δ*t* resulted in divergence of time series data. Therefore, after some trials, Δ*t* = 0.2 was selected and used throughout the paper.

Under the above model, we examined several *o* ^*i*^s as shown in Table 1. In order to examine network completion, WNT5A was modified by randomly adding *h* edges and deleting *k* edges and the resulting network was given as an initial network.

We evaluated the performance of the method in terms of the accuracy of the modified edges and the success rate. The accuracy is defined here by

(20) $$\begin{array}{c} \frac{h+k+\left|E_{\text{orig}}\right|-\left|E_{\text{orig}}\cap E_{\text{cmpl}}\right|}{h+k}, \end{array}$$

where *E*_orig_ and *E*_cmpl_ are the sets of edges in the original network and the completed network, respectively. This value takes 1 if all deleted and added edges are correct and 0 if none of the deleted and added edges is correct. For each (*h*, *k*), we took the average accuracy over a combination of 10 parameters (*a*_*j*_^*i*^s and *a*_*j*,*k*_^*i*^s) and 10 random modifications (i.e., addition of *h* edges and deletion of *k* edges to construct an initial network). The success rate is the frequency of the trials (among 10 × 10 trials) in which the original network was correctly obtained by network completion. The result is shown in Table 1. It is seen from this table that DPLSQ works well if the observation error level is small. It is also seen that the accuracies are high in the case of *h* = 0. However, no clear trend can be observed on a relationship between *h*, *k* values and the accuracies. It is reasonable because we evaluated the result in terms of the accuracy per deleted/added edge. On the other hand, it is seen that the success rate decreases considerably as *h* and *k* increase or the observation error level increases. This dependence on *h* and *k* is reasonable because the probability of having at least one wrong edge increases as the number of edges to be deleted and added increases. As for the computation time, the CPU time for each trial was within a few seconds, where we used the default values of *H* = *K* = 3. Although these default values were larger than *h*, *k* here, it did not cause any effects on the accuracy or the success rate. How to choose *H* and *K* is not a trivial problem. As discussed in Section 2.5, we cannot choose large *H* or *K* because of the time complexity issue. Therefore, it might be better in practice to examine several combinations of small values *H* and *K* and select the best result although how to determine the best result is left as another issue.

### 3.2. Inference Using Artificial Data

 We also examined DPLSQ for network inference, using artificially generated time series data. In this case, we used the same network and dynamics model as previously mentioned but we let *E* = *∅* in the initial network. Since the method was applied to inference, we let *H* = 0, *K* = 3, and *k* = 30. It is to be noted that deg^−^(*v*_*i*_) = 3 holds for all nodes *v*_*i*_ in the WNT5A network. Furthermore, in order to examine how CPU time changes as the size of the network grows, we made networks with 30 genes and 50 genes (with *k* = 90 and *k* = 150) by making 3 and 5 copies of the original networks, respectively.

Since the number of added edges was always equal to the number of edges in the original network, we evaluated the results by the average accuracy, which was defined as the ratio of the number of correctly inferred edges to the number of edges in the correct network (i.e., the number of added edges). We examined observation error levels of 0.1, 0.3, 0.5, and 0.7, for each of which we took the average over 10 trials using randomly generated different parameter values (i.e., *a*_*j*_^*i*^s and *a*_*j*,*k*_^*i*^s), where time series data were generated as in Section 3.1. The result is shown in Table 2, where the accuracy and the average CPU time (user time + sys time) per trial are shown for each case. It is seen from the table that the accuracy is high even for large networks if the error level is not high. It is also seen that although the CPU time grows rapidly as the size of a network increases, it is still allowable for networks with 50 genes.

We also compared DPLSQ with two well-known existing tools for inference of genetic networks, ARACNE [11, 12] and GeneNet [7, 8]. The former is based on mutual information and the latter is based on graphical Gaussian models and partial correlations. Computational experiments on ARACNE were performed under the same environment as that for DPLSQ, whereas those on GeneNet were performed on a PC with Intel Core i7-2600 CPU (3.40 GHz) with 16 GB RAM running under the Cygwin on Windows 7 because of the availability of the R platform on which GeneNet works. We employed datasets that were generated in the same way as for DPLSQ and default parameter settings for both tools.

Since both tools output undirected edges along with their significance values (or their probabilities), we selected the top *M* edges in the output where *M* was the number of edges in the original network and regarded {*v*_*i*_, *v*_*j*_} as a correct edge if either (*v*_*i*_, *v*_*j*_) or (*v*_*j*_, *v*_*i*_) was included in the edge set of the original network. As in Table 2, we evaluated the results by the average accuracy, that is, the ratio of the number of correctly inferred edges to the number of edges in the original network.

The result is shown in Table 3. Interestingly, both tools have similar performances. It is also interesting that the performance does not change much in each method even if the level of observation error changes. Readers may think that the accuracies shown in Table 3 are close to those by random prediction. However, these accuracies were much higher than those obtained by assigning random probabilities to edges, and thus we can mention that these tools outputted meaningful results.

It is seen from Tables 2 and 3 that the accuracies by DPLSQ are much higher than those by ARACNE and GeneNet even though both directions of edges are taken into account for ARACNE and GeneNet. However, it should be noted that time series data were generated according to the differential equation model on which DPLSQ relies. Therefore, we can only mention that DPLSQ works well if time series data are generated according to appropriate differential equation models. It is to be noted that we can use other differential equation models as long as parameters can be estimated by least-squares fitting.

As for computation time, both methods were much faster than DPLSQ. Even for the case of *N* = 50, each of ARACNE and GeneNet worked in less than a few seconds per trial. Therefore, DPLSQ does not have merits on practical computation time.

### 3.3. Inference Using Real Data

 We also examined DPLSQ for inference of genetic networks using real gene expression data. Since there is no gold standard on genetic networks and thus we cannot know the correct answers, we did not compare it with the existing methods.

**Figure 4:**
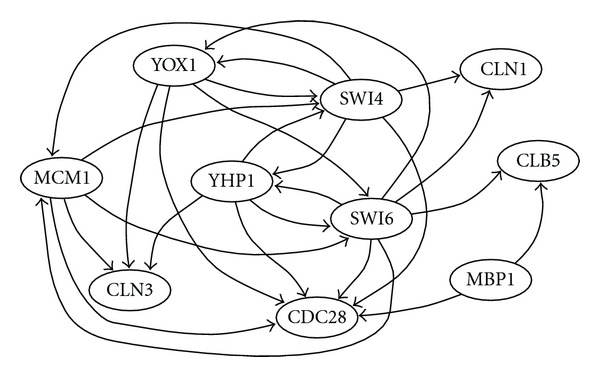
Structure of part of yeast cell cycle network.

We employed a part of the cell cycle network of * Saccharomyces cerevisiae* extracted from the KEGG database [18], which is shown in Figure 4. Although the detailed mechanism of the cell cycle network is still unclear, we used this network as the correct answer, which may not be true. Although each of (MCM1, YOX1, YHP1), (SWI4, SWI6), (CLN3, CDC28), (MBP1, SWI6) constitutes a protein complex, we treated them separately and ignored the interactions inside a complex because making a protein complex does not necessarily mean a regulation relationship between the corresponding genes.

As for time series data of gene expression, we employed four sets of times series data (alpha, cdc15, cdc28, elu) in [19] that were obtained by four different experiments. Since there were several missing values in the time series data, these values were filled by linear interpolation and data on some endpoint time points were discarded because of too many missing values. As a result, alpha, cdc15, cdc28, and elu datasets consist of gene expression data of 18, 24, 11, and 14 time points, respectively. In order to examine a relationship between the number of time points, and accuracy, we examined four combinations of datasets as shown in Table 4. We evaluated the performance of DPLSQ by means of the accuracy (i.e., the ratio of the number of correctly inferred edges to the number of added edges), where *K* = 3 and *k* = 25 were used. The result is shown in Table 4.

Since the total number of edges in both the original network and the inferred networks is 25 and there exist 9 × 10 = 90 possible edges (excluding self loops), the expected number of corrected edges is roughly estimated as

(21) $$\begin{array}{c} \frac{25}{90}\times 25=6.944\ldots , \end{array}$$

if 25 edges are randomly selected and added. Therefore, the result shown in Table 4 suggests that DPLSQ can do much better than random inference when appropriate datasets are provided (e.g., cdc15 only or cdc15+cdc28+alpha+elu). It is a bit strange that the accuracies for the first and last datasets are better than those for the second and third datasets because it is usually expected that adding more evidences results in more accurate networks. The reason may be that time series of cdc28 and alpha may contain larger measurement errors than those of cdc15 and elu, or some regulation rules that are hidden in Figure 4 may be activated under the conditions of cdc28 and/or alpha.

## 4. Conclusion

In this paper, we have proposed a network completion method, DPLSQ, using dynamic programming and least-squares fitting based on our previously proposed methodology of network completion [14]. As mentioned in Section 1, network completion is to make the minimum amount of modifications to a given network so that the resulting network is (most) consistent with the observed data. In our previous model [14], we employed the Boolean network as a model of networks and assumed that only expression or other values of one or a few nodes are observed. However, in this paper, we assumed that expression values of all nodes are observed, which correspond to gene microarray data, and regulation rules are given in the form of differential equations. The most important theoretical difference between this model and our previous model is that network completion can be done in polynomial time if the maximum indegree is bounded by a constant in this model whereas it is NP-hard in our previous model even if the maximum indegree is bounded by a constant. This difference arises not from the introduction of a least-squares fitting method but from the assumption that expression values of all nodes are observed.

It should also be noted that the optimality of the solution is not guaranteed in most of the existing methods for inference of genetic networks, whereas it is guaranteed in DPLSQ if it is applied to inference of a genetic network with a bounded maximum indegree. Of course, the objective function (i.e., minimizing the sum of squared errors) is different from existing ones, and thus this property does not necessarily mean that DPLSQ is superior to existing methods in real applications. Indeed, the result using real gene expression data in Section 3.3 does not seem to be very good. However, DPLSQ has much room for extensions. For example, least-squares fitting can be replaced by another fitting/regression method (with some regularization term) and the objective function can be replaced by another function as long as it can be computed by sum or product of some error terms. Studies on such extensions might lead to development of better network completion and/or inference methods.

## Figures and Tables

**Table 1:** Result on completion of WNT5A network, where the average accuracy is shown for each case.

No. deleted edges	No. added edges		Observation error level			
			0.1	0.3	0.5	0.7
*h* = 0	*k* = 1	Accuracy	0.990	0.910	0.730	0.410
		Success rate	0.99	0.91	0.73	0.41
*h* = 0	*k* = 2	Accuracy	1.000	0.955	0.670	0.395
		Success rate	1.00	0.91	0.42	0.17
*h* = 1	*k* = 0	Accuracy	0.990	0.850	0.470	0.240
		Success rate	0.99	0.85	0.47	0.24
*h* = 1	*k* = 1	Accuracy	0.995	0.845	0.405	0.210
		Success rate	0.99	0.71	0.11	0.02
*h* = 1	*k* = 2	Accuracy	0.983	0.843	0.470	0.190
		Success rate	0.95	0.58	0.11	0.00
*h* = 2	*k* = 0	Accuracy	1.000	0.795	0.440	0.215
		Success rate	1.00	0.67	0.18	0.01
*h* = 2	*k* = 1	Accuracy	0.996	0.833	0.453	0.223
		Success rate	0.99	0.53	0.05	0.01
*h* = 2	*k* = 2	Accuracy	1.000	0.862	0.517	0.285
		Success rate	1.00	0.56	0.03	0.01

**Table 2:** Result on inference of WNT5A network by DPLSQ.

		Observation error level			
		0.1	0.3	0.5	0.7
*n* = 10	Accuracy	1.000	0.966	0.803	0.620
	CPU time (sec.)	0.685	0.682	0.682	0.685

*n* = 30	Accuracy	0.995	0.914	0.663	0.443
	CPU time (sec.)	66.2	66.2	66.1	65.9

*n* = 50	Accuracy	0.999	0.913	0.613	0.392
	CPU time (sec.)	534.0	534.2	533.6	533.5

**Table 3:** Result on inference of WNT5A network using ARACNE and GeneNet, where the accuracy is shown for each case.

	Method	Observation error level			
		0.1	0.3	0.5	0.7
*n* = 10	ARACNE	0.523	0.523	0.523	0.526
	GeneNet	0.526	0.526	0.533	0.533

*n* = 30	ARACNE	0.332	0.328	0.326	0.326
	GeneNet	0.368	0.380	0.383	0.384

*n* = 50	ARACNE	0.308	0.312	0.310	0.391
	GeneNet	0.313	0.316	0.314	0.316

**Table 4:** Result on inference of a yeast cell cycle network.

Experimental conditions	Accuracy
cdc15	11/25
cdc15 + cdc28	8/25
cdc15 + cdc28 + alpha	8/25
cdc15 + cdc28 + alpha + elu	11/25
